# Supplementary material for: Interaction effects of the 5-HTT and MAOA-uVNTR gene variants on pre-attentive EEG activity in response to threatening voices
Source: Commun Biol. 2022 Apr 8;5:340. doi: 10.1038/s42003-022-03297-w (PMC8993814; doi:10.1038/s42003-022-03297-w)
Supplement: Supplementary file 2 — Description of Additional Supplementary Files [file 42003_2022_3297_MOESM2_ESM.pdf]

Description of Additional Supplementary Files

File name: Supplementary Data 1
Description: Source Data for Figure 2.

File name: Supplementary Data 2
Description: Source Data for Figure 3.

File name: Supplementary Data 3
Description: Source Data for Figure 4.
