# Supplementary material for: Interaction effects of the 5-HTT and MAOA-uVNTR gene variants on pre-attentive EEG activity in response to threatening voices
Source: Commun Biol. 2022 Apr 8;5:340. doi: 10.1038/s42003-022-03297-w (PMC8993814; doi:10.1038/s42003-022-03297-w)
Supplement: Supplementary file 6 — Reporting Summary [file 42003_2022_3297_MOESM6_ESM.pdf]

nature portfolio

Corresponding author(s): Chenyi Chen

Last updated by author(s): Jul 15, 2021

Reporting Summary

Nature Portfolio wishes to improve the reproducibility of the work that we publish. This form provides structure for consistency and transparency in reporting. For further information on Nature Portfolio policies, see our Editorial Policies and the Editorial Policy Checklist.

Statistics

For all statistical analyses, confirm that the following items are present in the figure legend, table legend, main text, or Methods section.

n/a	Confirmed	
☐	☒	The exact sample size (n) for each experimental group/condition, given as a discrete number and unit of measurement
☐	☒	A statement on whether measurements were taken from distinct samples or whether the same sample was measured repeatedly
☐	☒	The statistical test(s) used AND whether they are one- or two-sided<br>*Only common tests should be described solely by name; describe more complex techniques in the Methods section.*
☐	☒	A description of all covariates tested
☐	☒	A description of any assumptions or corrections, such as tests of normality and adjustment for multiple comparisons
☐	☒	A full description of the statistical parameters including central tendency (e.g. means) or other basic estimates (e.g. regression coefficient) AND variation (e.g. standard deviation) or associated estimates of uncertainty (e.g. confidence intervals)
☐	☒	For null hypothesis testing, the test statistic (e.g. F, t, r) with confidence intervals, effect sizes, degrees of freedom and P value noted<br>*Give P values as exact values whenever suitable.*
☒	☐	For Bayesian analysis, information on the choice of priors and Markov chain Monte Carlo settings
☐	☒	For hierarchical and complex designs, identification of the appropriate level for tests and full reporting of outcomes
☐	☒	Estimates of effect sizes (e.g. Cohen's d, Pearson's r), indicating how they were calculated

Our web collection on statistics for biologists contains articles on many of the points above.

Software and code

Policy information about availability of computer code

Data collection	Neuroscan 4.3 (Compumedics Ltd., Australia)
Data analysis	QIAamp DNA Mini Kit; Neuroscan 4.3 (Compumedics Ltd., Australia); Curry 8 (Compumedics Ltd., Australia); SPSS 17.0; IBM SPSS AMOS 23.0

For manuscripts utilizing custom algorithms or software that are central to the research but not yet described in published literature, software must be made available to editors and reviewers. We strongly encourage code deposition in a community repository (e.g. GitHub). See the Nature Portfolio guidelines for submitting code & software for further information.

Data

Policy information about availability of data

All manuscripts must include a data availability statement. This statement should provide the following information, where applicable:

- Accession codes, unique identifiers, or web links for publicly available datasets
- A description of any restrictions on data availability
- For clinical datasets or third party data, please ensure that the statement adheres to our policy

The datasets generated during and/or analysed during the current study are available from the corresponding author on reasonable request.

Field-specific reporting

Please select the one below that is the best fit for your research. If you are not sure, read the appropriate sections before making your selection.

☒ Life sciences ☐ Behavioural & social sciences ☐ Ecological, evolutionary & environmental sciences

For a reference copy of the document with all sections, see nature.com/documents/nr-reporting-summary-flat.pdf

Life sciences study design

All studies must disclose on these points even when the disclosure is negative.

Sample size	Sample size and power was estimated and calculated using GPower and Genetic Power Calculator.
Data exclusions	Data with values over two standard deviations around the mean were considered as outliers and excluded from further analysis.
Replication	To verify the reproducibility of our findings, we replicated all analyses within individual participants, internally validated analyses by generalizing across participants within the EEG/ERPs sample, and externally validated analyses by generalizing to an independent set of participants. Findings were consistent across within-individual, internal, and external validation.
Randomization	No randomization approach was applicable due to the use of within-subject design where all participants were exposed to the same experimental conditions.
Blinding	All participants were exposed to the same experimental conditions. Therefore, no blinding was necessary.

Reporting for specific materials, systems and methods

We require information from authors about some types of materials, experimental systems and methods used in many studies. Here, indicate whether each material, system or method listed is relevant to your study. If you are not sure if a list item applies to your research, read the appropriate section before selecting a response.

Materials & experimental systems

n/a	Involved in the study	
☒	☐	Antibodies
☒	☐	Eukaryotic cell lines
☒	☐	Palaeontology and archaeology
☒	☐	Animals and other organisms
☐	☒	Human research participants
☒	☐	Clinical data
☒	☐	Dual use research of concern

Methods

n/a	Involved in the study	
☒	☐	ChIP-seq
☒	☐	Flow cytometry
☒	☐	MRI-based neuroimaging

Human research participants

Policy information about studies involving human research participants

Population characteristics	The genetic and EEG part of this study comprised 140 adults (male/female ratio - 62/78), aged between 18 and 46 (23.82 ± 4.07) years. All participants were Han Chinese and right-handed. They participated in the study after providing written informed consent and were screened for major psychiatric illnesses (e.g., general anxiety disorder) by the Structured Clinical Interview for DSM-IV Axis I Disorders (SCID-I) and excluded if there was evidence of comorbid neurological disorders (e.g., dementia, seizures), history of head injury, and alcohol or substance abuse or dependence within the past five years. All participants were with normal hearing (pure tone average thresholds < 15 dB HL) at the time of testing. The subjects included in the data analysis were subdivided into sub-groups based on genotyping results of two candidate genes: MAOA-uVNTR (MAOA-high vs. MAOA-intermediate vs. MAOA-low) and 5HTT (SS vs. LL/LS). For 5HTT gene, participants possessing two copy of the S allele were included in the SS group (n=78) and those have homozygous for L or one copy of the L allele were included in the LS/LL group (n=62), respectively. For MAOA-uVNTR gene, since it is located on the X chromosome, the results from men and women were analyzed separately. Women can be classified as having high (H, n=29), intermediate (M, n=39) or low (L, n=10) MAOA-uVNTR activity but men can only be classified by having high (n=38) or low activity (n=24). The genotype frequencies for females were: L: 12.8%, M: 50%, H: 37.2%; for males they were: L: 38.7% and H: 61.3%.
Recruitment	Participants responded to fliers posted around National Yang-Ming University and expressed an interest in participating in EEG/ERPs experiments. All participants were screened to ensure they had normal normal bilateral peripheral hearing, and were not on any psychotropic medication.
Ethics oversight	Ethics Committee of National Yang-Ming University

Note that full information on the approval of the study protocol must also be provided in the manuscript.
